# Transcriptome profiling of *Cucumis melo* fruit development and ripening

**DOI:** 10.1038/hortres.2016.14

**Published:** 2016-04-27

**Authors:** Hong Zhang, Huaisong Wang, Hongping Yi, Wenqiang Zhai, Guangzhi Wang, Qiushi Fu

**Affiliations:** 1 Hami Melon Research Center, Xinjiang Academy of Agricultural Science, Urumqi 830091, China; 2 The Department of Cucurbitaceae Vegetables Genetics and Breeding, Institute of Vegetables and Flowers of the Chinese Academy of Agricultural Sciences, Beijing 100081, China

## Abstract

Hami melon (*Cucumis melo*) is the most important melon crop grown in the north-western provinces of China. In order to elucidate the genetic and molecular basis of developmental changes related to size, flesh, sugar and sour content, we performed a transcriptome profiling of its fruit development. Over 155 000 000 clean reads were mapped to MELONOMICS genome, yielding a total of 23 299 expressed genes. Of these, 554 genes were specifically expressed in flowers, and 3260 genes in fruit flesh tissues. The 7892 differentially expressed genes (DEGs) were related to fruit development and mediated diverse metabolic processes and pathways; 83 DEGs and 13 DEGs were possibly associated with sucrose and citric acid accumulation, respectively. The quantitative real-time PCR results showed that six out of eight selected candidate genes displayed expression trends similar to our DEGs. This study profiled the gene expression related to different growing stages of flower and fruit at the whole transcriptome level to provide an insight into the regulatory mechanism underlying Hami melon fruit development.

## Introduction

Melon (*Cucumis melo* L.) is an important annual diploid plant belonging to the Cucurbitaceae family. Melon plants produce large edible fruits that serve as an important dietary component worldwide.^1^ With increasing interest in biological properties and economic importance, melon has become an attractive model for studying valuable features, such as fruit ripening,^2^ sex determination^3,4^ and phloem physiology.^5^

The fruit development of medium maturing melon mainly includes four typical stages: young fruit, expanding, premature and mature. The open female flowers are ready for pollination only for about a day. Therefore, hand pollination is generally performed on the same day as flower opening. After pollination, cell division in the ovary is increased. Ten days after flowering (pollination; 10 DAF), ovarian epidermal cells stop proliferating leading to young fruit stage, with a small volume. Subsequently, following a rapid increase in the volume of young fruit, the cell diameters of the mesocarp, endocarp and placenta increase quickly resulting in fruit enlargement. Twenty days after flowering (20 DAF), the melon fruit enters the expanding stage, with the maximum fruit volume, and sugar content at a very low level. Subsequently, the fruit length is continuously increased, with rapidly increased sucrose content. Thirty days after flowering (30 DAF), the melon fruit enters a premature stage, cell expansion stops with maximum fruit volume and flesh firmness. The sucrose content consistently increases and reaches the maximum (60% of total sugar), with flesh turning soft in texture. Forty days after flowering (40 DAF), the melon fruit is considered fully mature or developed. At this stage, the sucrose content reaches the highest level, along with other nutritional components.

In recent years, a series of -omics studies on melon have been conducted. Dahmani-Mardas *et al.*^6^ compiled a list of candidate genes and isolated key mutants with enhanced fruit quality. Garcia-Mas *et al.*^7^ used the 454 pyrosequencing method to *de novo* sequence the melon double-haploid line DHL92 and obtained 375 Mb of assembled genome. In the same study, the melon genome sequence was phylogenetically compared with the genomes of cucumber and other plant species. Corbacho *et al.*^8^ used pyrosequencing to identify the candidate genes involved in the early induction of mature melon fruit abscission. Furthermore, the international cucurbit genomics initiative (ICuGI; http://www.icugi.org) conducted several genome-wide studies^7–9^ and provided valuable data to understand the mechanisms underlying sugar metabolism and mature fruit abscission in climacteric melon.

Hami melon is a melon cultivar geographically distributed in Xinjiang Turpan Hami area. As an ecological type, it mainly includes *C. melo* var *chandalak*, *ameri, cassaba* and *inodorus* cultivars. Hami melon is highly popular worldwide for its distinct flavor. ‘Flavor No. 4’ is a new cultivated medium maturing variety of Hami melon with unique sweet and sour taste. In order to better understand the processes of sucrose and organic acid accumulation in flesh, we performed a comprehensive transcriptome profiling of fruit flesh of ‘Flavor No. 4’. Our study may shed new light on the dynamics and differential expression of genes during fruit development and ripening of Hami melon.

## Materials and methods

### Plant materials

Melon plants (*C. melo* var *ameri*, cv ‘Flavor No. 4’) were grown in the greenhouse at the Institute of Vegetables and Flowers, Chinese Academy of Agricultural Sciences (Beijing, China) in 2013. Flowers were tagged at anthesis and one fruit was allowed to develop per plant. When melon flowers opened, the female flowers were hand-pollinated immediately. The fruits were collected 10, 20, 30 and 40 DAF. Female flower is the reproductive organ producing melon fruit and seeds. In order to comprehensively understand the process of Hami melon fruit development, the fully opened female flowers (0 DAF) were collected and included in this study. At each time point, three fruits/flowers were harvested from three different plants. Flesh mesocarp was obtained from the center-equatorial portion of each fruit. Tissue pieces were immediately frozen in liquid nitrogen and stored at −80 °C for the following analysis.

### High-performance liquid chromatography of sugar and organic acid content

The content of sugars and organic acids in fruit flesh were analyzed by high-performance liquid chromatography as previously described.^10^

### RNA isolation

Total RNA was extracted from each tissue using TRIzol kit (Invitrogen, Carlsbad, CA, USA) according to the manufacturer’s instructions. Subsequently, the RNA samples were treated with RQ1 DNase (Promega, Madison, WI, USA) to remove both double- and single-stranded DNA. The quality and quantity of the purified RNA were determined by measuring the absorbance at 260  nm/280  nm (A260/A280) on Smart Spec Plus Spectrophotometer (Bio-Rad, Philadelphia, PA, USA). RNA integrity was further verified by 1.5% agarose gel electrophoresis.

### Complementary DNA library preparation

For each sample, 10 mg of total RNA was used for RNA sequencing (RNA-seq) library preparation. Polyadenylated mRNAs were purified and concentrated with magnetic beads oligo (dT) (Invitrogen) before directional RNA-seq library preparation. Purified mRNAs were fragmented at 95 °C followed by end-repair and 5ʹ adapter ligation. The reverse transcription was performed with reverse transcription primer harboring 3ʹ adapter sequence and randomized hexamer. The complementary DNAs were purified and amplified and PCR products corresponding to 200–500 bp were purified, quantified and stored at −80° C until used for sequencing.

### Whole-transcriptome sequencing

For high-throughput sequencing, the prepared libraries were subjected to Illumina Hiseq 2000 system 100 nucleotides (nt) pair-end sequencing (ABlife, Inc., Wuhan, China).

### RNA-seq raw data clean and alignment statistics

First, raw reads containing more than 2-N bases were discarded, and the remaining reads were processed by clipping adapters. Low-quality bases were removed. Reads less than 16 nt were also dropped. FASTX-Toolkit (Version 0.0.13) was used to obtain the clean reads. Subsequently, clean reads were aligned to MELONOMICS genome sequence (https://melonomics.net/) by Tophat2.^11^ On the basis of the genome location of the reads, aligned reads containing more than one genome location were discarded due to their ambiguous location. Uniquely localized reads were used to calculate the read number and RPKM (reads per kilobase and per million) value for each gene, according to their genome location. Other statistical results, such as gene coverage and depth, read distribution around start codon and stop codon, were also obtained. RNA-seq data were deposited in the NCBI Sequence Read Archive (NCBI SRA SRX641227).

### Hierarchical clustering analysis

Hierarchical clustering was performed to calculate the cluster of genes set by Cluster 3.0 software. Heat maps showing expression profiles (log_2_ RPKM) were generated using Java Tree View (http://jtreeview.sourceforge.net, Baryshnikova Lab, Princeton University, Princeton, NY, USA).

### Data analysis of transcriptome profiles of differentially expressed genes

Differentially expressed genes (DEGs) between two samples were analyzed using edge R,^12^ one of R packages. For each gene, the *P-*value <0.01 and foldchange >2 or <0.5 were considered as the significant threshold. All DEGs were mapped to terms in Gene Ontology (GO) and Kyoto Encyclopedia of Genes and Genomes (KEGG) databases. For GO analysis, Hami melon-expressed genes were aligned to the NR database with a 1e^−5^ *E-*value threshold, and the alignment were assigned GO functional descriptions by Blast2GO software.^13^ For KEGG analysis, Fisher’s exact test was used to define the enrichment of each GO term and KEGG pathway. Other statistical results were obtained using R software.

### Quantitative real-time PCR analysis

To validate the accuracy of our transcriptome profiling, the gene expression of eight randomly selected candidate genes was evaluated by quantitative real-time PCR. Total RNA was extracted from female flowers at 0 DAF and fruit flesh at 10 DAF, 20 DAF, 30 DAF and 40 DAF with TRIzol kit (Invitrogen). The total RNA was reverse transcribed using QuantiTect Reverse Transcription kit (Qiagen, Valencia, CA, USA) according to the manufacturer’s protocol. Actin was used as an internal control. Real-time monitoring of PCR was performed with ABI 7500 Real Time System and SYBR GREEN PCR Master Mix (Applied Biosystems, Grand Island, NY, USA). Reactions were performed at 94 °C for 2 min, and then cycled at 95 °C for 30 s, 48–54 °C for 30 s, 72 °C for 30 s for 35 cycles and then 72 °C for 10 min. Each assay was performed in triplicates. Data analysis was performed using the 2^−ΔΔCt^ method described previously.^14^

## Results

### Fruit size, sugar and organic acid content of ‘Flavor No. 4’ during fruit development

**Figure 1:**
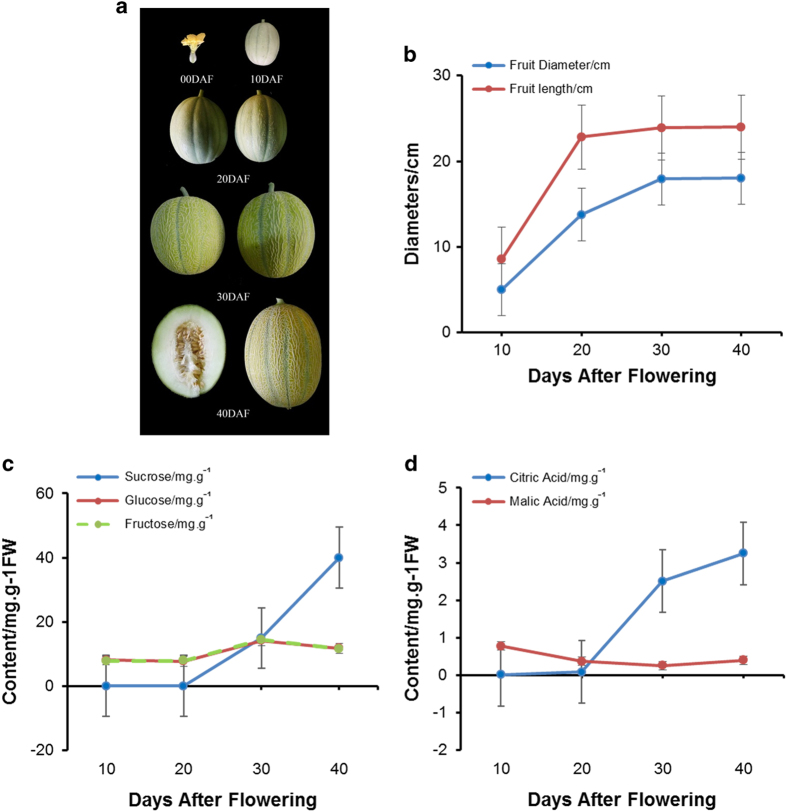
(**a**) The opening female flower and melon fruit of ‘Flavor No. 4’ at different developmental stages. (**b**) The size (length, diameter) of ‘Flavor No. 4’ fruit at 10, 20, 30 and 40 days after flowering (DAF). Data are the means of three measurements and s.e. values. (**c**) Soluble sugars (sucrose, glucose and fructose) concentrated in ‘Flavor No. 4’ fruit flesh at 10, 20, 30 and 40 DAF. Data represent the means of three extractions and s.e. values. (**d**) Organic acid (citric acid and malic acid) concentration in ‘Flavor No. 4’ fruit flesh at 10, 20, 30 and 40 DAF. Data represent the means of three extractions and s.e. values.

‘Flavor No. 4’, a medium maturing variety of Hami melon, was used in the present study. It belongs to the *C. melo* var *ameri* (pang) Greb. It is a Hami melon cultivar that produces short oval shape, medium size and yellow skin melon with 10 green longitudinal grooves on the surface. This type of melon contains white juicy flesh with unique sweet and sour taste (Figures 1a, c and d). The fruit size, sugar and organic acid content of fruit flesh at four typical developmental stages were examined. At 10 DAF, the volume of melon fruit was still small; from 10 to 20 DAF, the fruit diameter and length both increased rapidly; from 20 to 30 DAF, the fruit diameter slightly increased, the fruit length continuously rapidly increased; from 30 to 40 DAF, the fruit volume increased to the maximum (Figures 1a and b). The sucrose, glucose and fructose contents in the fruit flesh were examined by high-performance liquid chromatography. As shown in Figure 1c, the glucose and fructose content remained at a low level during fruit development, whereas the sucrose content increased markedly from 20 to 40 DAF, reaching its highest level at 40 DAF. This finding suggested that rapid sucrose accumulation at the medium and late developmental stages contributed to the sweet taste of fruit flesh. The contents of citric acid (CA) and malic acid were detected by high-performance liquid chromatography. As shown in Figure 1d, the content of CA increased quickly from 20 to 40 DAF, and the CA content during 20–30 DAF increased even faster than that at 30–40 DAF. However, the malic acid level decreased slightly during fruit development indicating that the rapid accumulation of CA in the medium and late developmental stages determined the sour flavor of fruit flesh.

### Transcriptome profiles of the female opening flowers and the flesh mesocarp

RNA-seq was carried out on the complementary DNA libraries derived from the female opening flowers (0 DAF) and the flesh mesocarp of fruit at 10, 20, 30 and 40 DAF. The processed clean reads were aligned to MELONOMICS genome sequence. An overview of the results is displayed in Table 1. The majority of reads from each sample (76.07–94.48%) were successfully aligned, except that the alignment rate for sample 10 DAF was relatively low (52.19%). We found the reads from sample 10 DAF contained many 20–40 nt short reads, which cannot be mapped to the reference genome. Between 12 and 24 million reads per sample were uniquely aligned to the MELONOMICS genome and over 18 000 expressed genes were detected in each sample (Table 1). On the whole, 23 299 of the 27 427 annotated genes (84.95%) were detected in flower and flesh mesocarp samples.

**Figure 2:**
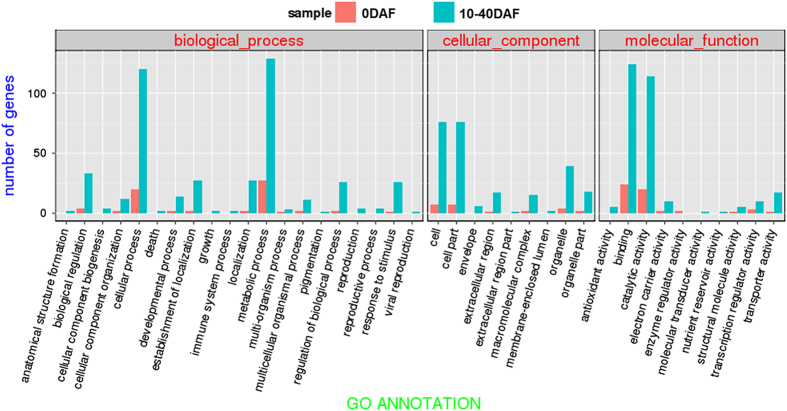
Functional classification of the genes specifically expressed in ‘Flavor No. 4’ female flower or fruit flesh within the category of biological process, molecular function and cellular component.

We found that 20 206 and 22 922 genes were expressed in female flowers (0 DAF) and fruit flesh (10–40 DAF), respectively, with 554 genes specifically expressed in 0 DAF (Figure 2), and 3260 genes specifically expressed in fruit at 10–40 DAF (Figure 2). Hami melon-expressed genes were assigned to GO functional descriptions based on their sequence matches to known proteins in the NR database. Hami melon-expressed genes were aligned to the NR database with a 1e^−5^ *E-*value threshold, and the alignment were assigned GO functional descriptions by Blast2GO software.^13^ In female flowers, 2822 genes were assigned with at least one GO term in the biological process category, 2890 genes in the molecular function category and 1759 in the cellular component category. Although in fruit flesh, 2960 genes were assigned with at least one GO term in the biological process category, 3043 genes in the molecular function category and 1841 in the cellular component category. Cellular process, cell part and binding are the most abundant GO slims within the biological process, cellular component and molecular function categories, respectively. Cellular process (2189 genes in female flowers, 2289 genes in fruit flesh), metabolism process (2174 genes in female flowers, 2276 genes in fruit flesh) and biological regulation (594 genes in female flowers, 623 genes in fruit flesh) were among the most highly represented groups within the biological process category, indicating both the female flower and fruit flesh tissue was undergoing extensive metabolic activities. In addition, genes involved in other important biological processes such as establishment of localization, regulation of biological process and response to stimulus were also identified. GO terms were also assigned to the specifically expressed genes in female flowers and fruit flesh. As shown in Figure 2, compared with fruit flesh tissues, enzyme regulator activity was represented within the molecular function category of flower tissues. In the fruit flesh, antioxidant, molecular transducer and nutrient reservoir activities within the molecular function category; anatomical structure, cellular component organization, growth, immune system within the biological process; and envelope, extra cellular region and membrane-enclosed lumen within the cellular component were the unique groups compared with the flower tissues. This finding suggested that the genes categorized in these groups may exert tissue-specific function during plant development.

**Figure 3:**
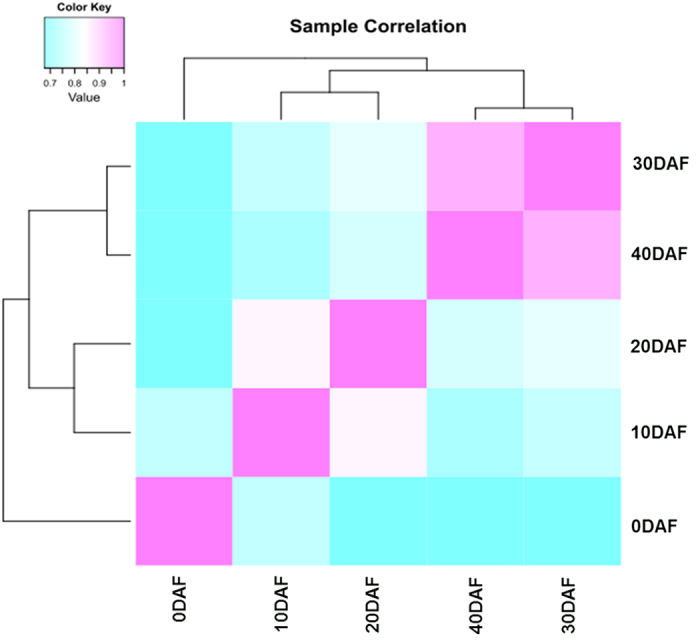
Hierarchical clustering analysis of gene expression levels in each of the five samples (0, 10, 20, 30 and 40 days after flowering (DAF)). Shades of blue indicate lowered expression, relative to the mean across samples, whereas shades of pink indicate higher expression relative to the mean.

Hierarchical clustering analysis was used to investigate the expression patterns of genes in female flowers and fruit flesh. As shown in Figure 3, the 10 DAF was assigned to the adjacent node of 20 DAF, 30 DAF was grouped into the same cluster of 40 DAF, while 0 DAF showed distinct gene expression pattern. This finding demonstrated tissue-specific gene expression of flower different from fruit flesh. During fruit development, similarities in gene expression pattern were observed between 10 and 20 DAF, and 30 and 40 DAF, suggesting that the gene expression was significantly altered from 20–30 DAF, with implications for fruit development.

### DEGs during fruit development

A total of 7892 genes showed significant upregulation or downregulation (*P*-value <0.01, foldchange >2 or <0.5) during fruit development.

**Figure 4:**
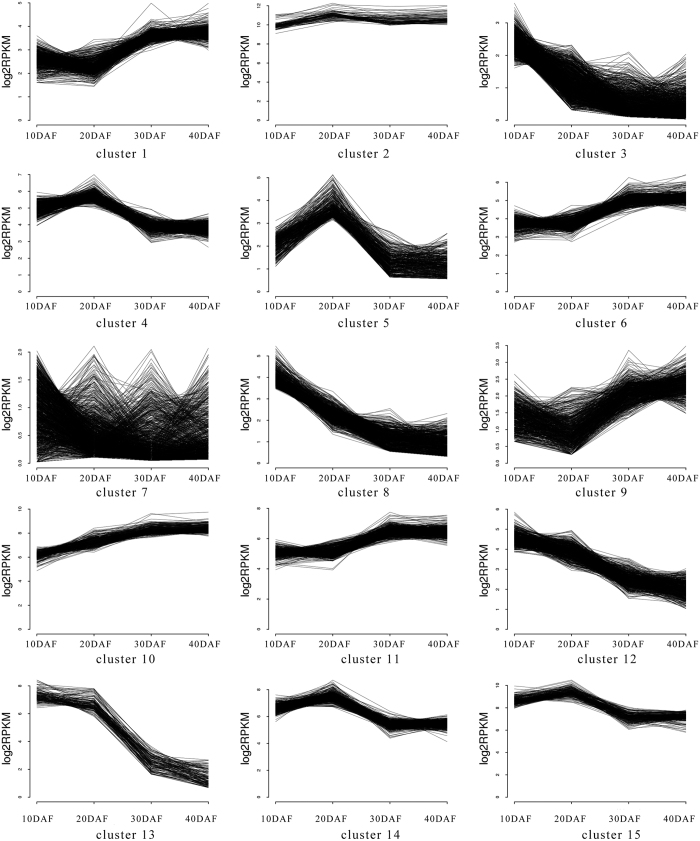
Gene expression pattern obtained by *K*-means clustering. Differentially expressed genes (DEGs) in fruit flesh were categorized into 15 clusters depending on their expression during fruit development. Levels of gene expression were represented along the *y* axis as log_2_ RPKM, and the stages of fruit development were represented along the *x* axis as 10, 20, 30 and 40 days after flowering (DAF).

On the basis of similar kinetic patterns of expression, all 7892 DEGs were classified into 15 types of clusters (Figure 4). The gene expression patterns of cluster 1 (259 DEGs), cluster 6 (233 DEGs), cluster 9 (204 DEGs) and cluster 11 (263 DEGs) exhibited similar changes during fruit development. The gene expression levels showed a rapid increase from 20 to 30 DAF, but changed little between 10 and 20 DAF and between 30 and 40 DAF. Genes in this group may function in the middle stages of fruit development. The gene expression patterns of cluster 4 (207 DEGs), cluster 13 (19 DEGs), cluster 14 (188 DEGs) and cluster 15 (107 DEGs) were similar. The gene expression levels showed rapid decrease from 20 to 30 DAF, but changed little during the rest of the developmental stages. Genes in this group may also have a role in the middle stages of fruit development. The gene expression patterns of cluster 3 (288 DEGs), cluster 8 (195 DEGs) and cluster 12 (208 DEGs) displayed a continuous decline during fruit development. These genes may have an important role at the young fruit stage. The expression levels of genes in cluster 2 (37 DEGs) and cluster 10 (192 DEGs) were maintained at a high level during the entire development. The gene expression levels in cluster 2 were altered minimally at different development stages. The gene expression levels in cluster 10 showed a slight increase during fruit development. Genes in these two clusters may exert a fundamental role in the fruit developmental process. Genes in cluster 5 (106 DEGs) were specifically highly expressed at 20 DAF, indicating a close relationship with fruit expansion. Genes in cluster 7 (394 DEGs) had a relatively low expression in the fruit developmental process.

### Analysis of the candidate genes involved in fruit development and ripening

To systematically assess the biological functions of the candidate genes, KEGG enrichment analysis was performed. The candidate DEGs were assigned to 16 KEGG pathway (*P*-value ⩽0.05) with significant enrichment.

### Genes mediating sugar metabolism

Eighty-three DEGs were found to be associated with sucrose metabolism, belonging to 13 different gene expression clusters, of which, 12 genes were associated with sugar metabolism in melon fruit^7,15^ (ICuGI v4; http://www.icugi.org). In the current study, the sucrose phosphate synthase 2 (*CmSPS2*) (MELO3C020357) increased markedly with fruit maturation and reached the highest levels in premature fruit. Three sucrose synthases (*CmSUS1, CmSUS2* and *CmSUS-LIKE1*) (MELO3C015552, MELO3C025101 and MELO3C001956) showed different expression patterns during fruit development and ripening. The expression level of *CmSUS1* was slightly decreased from the young fruit stage to expanding stage, and then increased rapidly to the highest level at premature stage, with a slight decrease in the mature fruit. The expression level of *CmSUS2* was the highest in young fruit, and consistently decreased in the following developmental stages. The expression level of *CmSUS-LIKE1* was relatively low in young fruits and was continuously decreased during fruit development. Two hexokinases (*CmHK2* and *CmHK3*) (MELO3C015416 and MELO3C007677) were differentially expressed during fruit development of Hami melon. The expression level of *CmHK2* was similar in the young fruit and expanding stages, increased rapidly at premature stage, and slightly decreased in the ripe fruit. The *CmHK3* expression was low in young fruit and decreased during fruit development. The expression level of fructokinase 3 (*CmFK3*) (MELO3C022452) gradually increased during fruit development, reaching the highest value in the ripe fruit. The expression level of acid invertases 2 (*CmAIN2*) (MELO3C005363) showed the opposite trend and declined during fruit development. Two cell wall invertases (*CmCIN2* and *CmCIN3*) (MELO3C024383 and MELO3C010751) were differentially expressed during fruit development. The expression level of *CmCIN3* was continuously increased, peaking in the premature stage, and declined in the ripe fruit. The expression of *CmCIN2* was low during fruit development. The expression level of phosphoglucose isomerase cyt (*CmPGIcyt*) (MELO3C010936) was rapidly increased in the early developmental stages, peaking at the late developmental stages. The expression level of alpha-galactosidase 2 (*GAL2*) (MELO3C011771) was the highest in young fruit, consistently declined during fruit development and reached its lowest value in ripe fruit. Subsequently, we compared the gene expression patterns of these 12 genes in our transcriptome profiles from the previous study^14^ (Table 2), most of which showed varying trends.

In addition to the above 12 DEGs, 71 DEGs were also found associated with sugar metabolism pathways during Hami melon fruit development and ripening. Their expression patterns were categorized into 13 clusters.

### Genes involved in citrate acid cycle

The 13 DEGs identified in this study were possibly related to CA cycle during Hami melon fruit development, and were categorized into 9 expression pattern clusters (Table 3). Three phosphoenolpyruvate carboxykinases (*PCK1, PCK2* and *PCK3*) (MELO3C007687, MELO3C018994 and MELO3C003491) were differentially expressed during fruit development. The expression level of *PCK1* was the highest in the young fruit, drastically declined to the lowest level in ripe fruit. The expression level of *PCK2*, on the contrary, slightly increased in the early and middle stages of fruit development, and then drastically increased at the late stages of fruit development. The expression level of *PCK3* remained the same in young fruit and expanding stages, and then rapidly decreased in the middle developmental stage, reaching a low at premature and mature stages. Two cytosolic NADP+-dependent isocitrate dehydrogenases (*cICDH1* and *cICDH2*) (MELO3C005968 and MELO3C021563) were differentially expressed during fruit development. The *cICDH2* expression level increased markedly in the middle of fruit development, reached the highest level in premature fruit and slightly decreased in the mature fruit, whereas the expression of *cICDH1* was maintained at a low level throughout the fruit development process. Two lipoamide dehydrogenase 1 (*LPD1*) (MELO3C021276 and MELO3C021247) genes showed similar changes during fruit development, with increased expression levels during fruit development and the highest value in ripe fruit. Two malate dehydrogenase (*MD1, MD2*) (MELO3C017175, MELO3C005968) genes showed the opposite trend. Although the expression level of *MD1* increased, the expression level of *MD2* decreased during fruit development. The expression level of aconitate 1 (*ACO1*) (MELO3C007942) was markedly increased in the middle stage of fruit development, and reached the highest value in ripe fruit. The expression level of 2-oxoglutarate dehydrogenase (*OGDH*) (MELO3C025454) was the highest in young ripe fruit, and drastically declined in the expanding stage and reached the lowest value in ripe fruit. Pyruvate dehydrogenase (*PD*) (MELO3C023666) highly expressed in young fruit, declined to the lowest level in premature fruit and slightly increased in ripe fruit.

### Verification of DEGs during Flavor No. 4 Hami melon fruit development

**Figure 5:**
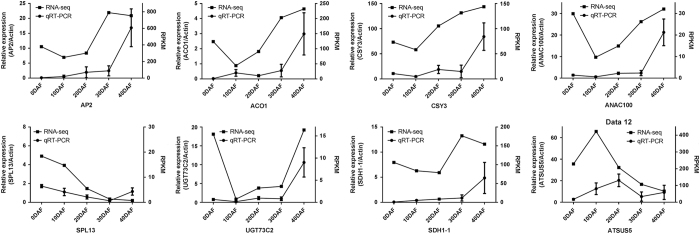
Quantitative real-time PCR (qRT-PCR) analysis of DEGs related to fruit development. The left *y* axis represents the relative gene expression levels analyzed by qRT-PCR and the right *y* axis represents the RPKM value analyzed by RNA sequencing (RNA-seq). Bars represent the s.e. (*n*=3).

In order to verify the expression patterns of DEGs involved in fruit development, eight genes were randomly selected for quantitative real-time PCR analysis. Three genes (*ACO1, CSY3* and *SDH1-1*) were involved in citrate cycle, two genes (*SUS5* and *UGT73C2*) in carbohydrate metabolism, and three were transcription factors (*AP2, ANAC100* and *SPL13*). As shown in Figure 5, although the exact gene expression pattern of the selected genes at several time points varied between RNA-seq and quantitative real-time PCR analysis, their altered expression was similar. These results confirmed the accuracy of our transcriptome profiling.

## Discussion

The RNA-seq study detected 23 299 genes expressed in female flowers and fruit flesh during ‘Flavor No. 4’ Hami melon development. Of these genes, 554 were specifically expressed in female opening flowers and 3260 were specifically expressed in fruit flesh tissues. These genes were assigned to various functional categories and may exert tissue-specific function during plant development. Hierarchical clustering analysis revealed a fundamental change in gene expression pattern between expanding and premature stages, with significant role in fruit development and ripening. A total of 7892 DEGs were identified during fruit development, and categorized into 15 different expression clusters. The candidate DEGs were assigned to 16 KEGG pathways with significant enrichment.

Hierarchical clustering analysis revealed a fundamental change in gene expression patterns between expanding and premature stages. Among the 7892 DEGs, 259 DEGs in cluster 1, 233 DEGs in cluster 6, 204 DEGs in cluster 9 and 263 DEGs in cluster 11 showed a rapid increase in expression from expanding to premature stages, with little change during the rest of development stages. The 207 DEGs in cluster 4, 19 DEGs in cluster 13, 188 DEGs in cluster 14 and 107 DEGs in cluster 15 showed a rapid decrease in expression from expanding to premature stage. In addition, 106 DEGs in cluster 5 were highly expressed in the expanding stage. All these DEGs probably had a critical role in Hami melon fruit development.

In the KEGG enrichment analysis, the candidate DEGs associated with fruit development were assigned to 16 functional pathways with significant enrichment. Of these, the plant hormone signal transduction pathway mediated fruit ripening.^16^ In this pathway, the ethylene and fruit-specific transcription factors together with epigenome modification mediated the transition of fruit to the ripening stage.^17^ In this study, DEGs in plant hormone signal transduction pathway showed different gene expression patterns during the fruit development process, suggesting a role in the regulation of fruit development.

Melons are consumed for their sweet fruit,^18^ characterized by the accumulation of sucrose during the late stage of fruit development.^19–23^ In this study, the sucrose content increased markedly from fruit expansion to mature stage, and reached its highest level in ripe fruit, demonstrating the rapid accumulation of sucrose in the medium and late developmental stages in ‘Flavor No. 4’ Hami melon fruit. Sucrose accumulation in the melon fruit mesocarp is a developmentally regulated process related to metabolic transition.^20,24–31^ The detailed sucrose metabolic pathway in melon has been introduced in a previous study.^15^ In the present study, 83 DEGs were found to mediate sugar metabolism. Of these, 12 genes have been related to sucrose accumulation in melon fruit.^7,15^ We compared the expression pattern of the 12 genes in this study with the transcriptome profiles in a previous study.^15^ Our results showed three genes, *Hexokinase3*, *CmAIN2* and *CmPGIcyt,* with similar alterations during fruit development, and five genes, *CmSPS2*, *CmSUS2, CmFK3, Hexokinase 2* and *GAL2,* showing opposite trends, while the expression of *CmSUS-LIKE1, CmCIN2* and *CmCIN3* was not detected in Dai’s study (Table 3). Different melon varieties and growing conditions led to differences in gene expression pattern in different studies.

In sweet melons, sucrose is the primary component determining fruit quality.^18,19^ However, a few varieties accumulate high levels of acid in melon fruit.^32–35^ Breeders combine the high acidity and high sugar traits of melons to develop dessert cultivars.^36^ ‘Flavor No. 4’ Hami melon is one such cultivar with unique sweet and sour taste, with CA as the major organic acid in its ripe fruit.^37^ In this study, the CA content in fruit flesh showed a rapid increase from expanding to mature stages, indicating the CA accumulation in the late developmental stage. Generally, the acid content of a fruit is determined by the balance of acid synthesis and degradation. Organic acid metabolism in fruits is a complex physiological process. As described before,^36^ *PEPC* catalyzes the synthesis of oxaloacetic acid (OAA) from PEP, *MD* catalyzes malic acid synthesis from OAA, both of which enter the tricarboxylic acid cycle. Citrate synthase (CS) catalyzes CA synthesis using acetyl coenzyme A (Ac-CoA) and OAA. CA enters the cytoplasm, and accumulates in the vacuoles with the help of H^+^ pump active transport carrier. As the CA level in vacuoles accumulates to a high level, it is released into the cytoplasm. In the cytoplasm, cytaconitase (*ACO*) converts citrate to isocitrate, which is unstable, and is converted by isocitrate dehydrogenase (NADP–IDH) to generate ketone glutaric acid. In different fruit species, different enzymes catalyze organic acid metabolism. Phosphoenolpyruvate carboxylase (*PCK*), *CS*, aconitase (*ACO*) and isocitrate dehydrogenase (*cICDH*) in grape,^38^ pineapple,^39^ peach^38^ and lemon,^40–42^ respectively, and *MD* in apple, strawberry and grape^43,44^ have a critical role in the accumulation of organic acids in fruits. In the present study, we found that 13 DEGs possibly mediated CA accumulation. The *PCK* and *MD* were involved upstream of CA synthesis. PCK is the key enzyme that catalyzed the conversion of PEP to OAA, and MD catalyzed conversion of OAA to malic acid, which entered the CA cycle. In this study, the expression level of *PCK1* was the highest in young fruit, drastically declined to its lowest level in ripe fruit. The expression level of *PCK2* was slightly increased in the early and middle stages of fruit development, drastically increased at the late stage of fruit development. The *PCK3* expression was rapidly decreased in the middle stage, to reach a low level at premature and mature stages. The MD1 expression level increased during the fruit development, while the MD2 level decreased during fruit development. The rapid increase in *PCK2 and MD1* may contribute to CA accumulation in mature fruit. Aconitase 1 (*ACO1*) and *cytosolic* NADP^+^-dependent isocitrate dehydrogenase (*cICDH*) mediate downstream CA synthesis. *ACO1* has an important role in catalyzing isocitrate formation, resulting in CA degradation.^45^ In our study, the expression level of aconitate 1 (*ACO1*) (MELO3C007942) was markedly increased in the middle stage of fruit development, and reached the highest value in ripe fruit. The *cICDH2* increased markedly in the middle stage of fruit development, reaching the highest level in premature fruit and slightly decreased in the mature fruit, whereas the expression of *cICDH1*was maintained at a low level throughout the fruit development process. The upstream enzymes of citrate acid cycle work synergistically to accumulate CA. Excessive cellular levels of CA activate the downstream enzymes, which initiate its degradation.

We identified the DEGs mediating various pathways in fruit development and ripening of ‘Flavor No. 4’. Our results provide valuable insights in elucidating the molecular mechanism of sucrose and CA accumulation during melon fruit development.

## Figures and Tables

**Table 1:** Summary of RNA-seq data collected at five important stages of fruit development

*Category*	*0 DAF*	*10 DAF*	*20 DAF*	*30 DAF*	*40 DAF*
Input reads	25 310 332	25 757 533	20 324 207	24 080 391	19 762 651
Aligned to genome	23 735 470 (93.78%)	13 442 267 (52.19%)	15 459 847 (76.07%)	22 746 535 (94.48%)	18 036 713 (91.27%)
Uniquely aligned to genome	23 449 265 (92.65%)	12 683 194 (49.24%)	15 215 812 (74.87%)	22 509 389 (93.48%)	17 814 425 (90.14%)
Detected genes	20366	21447	19815	18468	18484

Abbreviations: DAF, days after flowering; RNA-seq, RNA sequencing.

Read counts in the middle section are expressed in numbers (left) or as a percentage of the input reads processed (right) for each sample.

**Table 2:** The 12 differentially expressed genes associated with sugar metabolism pathway in melon fruit (Dai *et al;*^15^ Garcia-Mas *et al.*^7^) (ICuGI v4; http://www.icugi.org)

*Gene ID*	*Gene description*	*Short form*	*Expression patterns from the previous study (Dai* et al.^15^)	*Expression patterns in current study*
MELO3C020357	Sucrose-P synthase 2	CmSPS2	Only weakly expressed during fruit development with no obvious pattern of expression	Increased markedly with fruit maturation and reach highest levels in premature fruit
MELO3C015552	Sucrose synthase 1	CmSUS1	Very strongly expressed in the young fruit. Subsequently, decline rapidly and non-expressed in the maturing fruit	Slightly decreased from the young fruit stage to expanding stage, increased rapidly and reached the highest level at premature stage, and slightly decreased in the mature fruit.
MELO3C025101	Sucrose synthase 2	CmSUS2	Low levels of expression throughout fruit development	Highest in young fruit, and consistently decreased in the following developmental stages
MELO3C001956	New putative sucrose synthase	CmSUS-LIKE1	No	Low in young fruit and continuously decreased during fruit development
MELO3C022452	Fructokinase 3	CmFK3	Maximal expression in the young fruit, declining during fruit development	Rising during fruit development, reaching the highest value in ripe fruit
MELO3C015416	Hexokinase 2	CmHK2	Not observed at all	No change from young fruit stage to expanding stage, rising quickly at premature stage, and slightly decreasing in ripe fruit
MELO3C007677	Hexokinase 3	CmHK3	Very weakly expressed	Low in young fruit and decreased during fruit development
MELO3C005363	Acid invertase 2	CmAIN2	Highly expressed in young developing fruit, and subsequently declined substantially at the sucrose accumulation stage	Decreased during fruit development, lowest in ripe fruit
MELO3C024383	Cell wall invertase 2	CmCIN2	No	Low level during fruit development
MELO3C010751	Cell wall invertase 3	CmCIN3	No	Continuously increased, peaking at premature stage, and declining in ripe fruit
MELO3C010936	Phosphoglucose isomerase cyt	CmPGIcyt	Moderate, and increasing during fruit development	Rapidly increased at early developmental stages, peaking at the late developmental stages
MELO3C011771	Alpha-galactosidase 2	GAL2	Most highly expressed in young fruit, and highly expressed in mature fruit	Highest in young fruit, consistently declined during fruit development to reach its lowest value in ripe fruit

**Table 3:** The 13 differentially expressed genes possibly related to citrate cycle

*Gene ID*	*Genes description*	*Gene expression patterns*	*Reference*	*Crop*
MELO3C017175	Malate dehydrogenase	1	Taureilles-Saurel;^43^ Iannetta *et al.*^44^	Apple, strawberry
MELO3C021276	Lipoamide dehydrogenase 1	1	No	
MELO3C007687	Phosphoenolpyruvate carboxykinase 1	3	Diakou *et al.*;^46^ Sadka *et al.*^40–42^	Grape, lemon
MELO3C005968	Cytosolic-NAD-dependent malate dehydrogenase 1	3	Taureilles-Saurel;^43^ Iannetta *et al.*^44^	Apple, strawberry
MELO3C023666	Pyruvate dehydrogenase	4	No	
MELO3C021247	Mitochondrial lipoamide dehydrogenase 1	6	No	
MELO3C011481	Unknown	7	No	
MELO3C025454	2-oxoglutarate dehydrogenase	8	No	
MELO3C018994	Phosphoenolpyruvate carboxykinase 1	10	Diakou *et al.*;^46^ Sadka *et al.*^40–42^	Grape, lemon
MELO3C025076	Cytosolic NADP+-dependent isocitrate dehydrogenase	10	Diakou *et al.*;^46^ Sadka *et al.*^40–42^	Grape, lemon
MELO3C007942	Aconitase 1	10	Diakou *et al.*;^46^ Sadka *et al.*^40–42^	Grape, lemon
MELO3C021563	Cytosolic NADP+-dependent isocitrate dehydrogenase	11	Diakou *et al.*;^46^ Sadka *et al.*^40–42^	Grape, lemon
MELO3C003491	Phosphoenolpyruvate carboxykinase 1	14	Diakou *et al.*;^46^ Sadka *et al.*^40–42^	Grape, lemon
